# Cell scale host-pathogen modeling: another branch in the evolution of constraint-based methods

**DOI:** 10.3389/fmicb.2015.01032

**Published:** 2015-10-06

**Authors:** Neema Jamshidi, Anu Raghunathan

**Affiliations:** ^1^Institute of Engineering in Medicine, University of CaliforniaSan Diego, La Jolla, CA, USA; ^2^Department of Radiological Sciences, University of California, Los AngelesLos Angeles, CA, USA; ^3^Chemical Engineering Division, National Chemical LaboratoryPune, India

**Keywords:** constraint-based model, host-pathogen, optimization methods, mathematical models, omics-technologies, tuberculosis, salmonella typhimurium, flux balance analysis

## Abstract

Constraint-based models have become popular methods for systems biology as they enable the integration of complex, disparate datasets in a biologically cohesive framework that also supports the description of biological processes in terms of basic physicochemical constraints and relationships. The scope, scale, and application of genome scale models have grown from single cell bacteria to multi-cellular interaction modeling; host-pathogen modeling represents one of these examples at the current horizon of constraint-based methods. There are now a small number of examples of host-pathogen constraint-based models in the literature, however there has not yet been a definitive description of the methodology required for the functional integration of genome scale models in order to generate simulation capable host-pathogen models. Herein we outline a systematic procedure to produce functional host-pathogen models, highlighting steps which require debugging and iterative revisions in order to successfully build a functional model. The construction of such models will enable the exploration of host-pathogen interactions by leveraging the growing wealth of omic data in order to better understand mechanism of infection and identify novel therapeutic strategies.

## Why constraint-based modeling for host-pathogen interactions?

**Figure 1:**
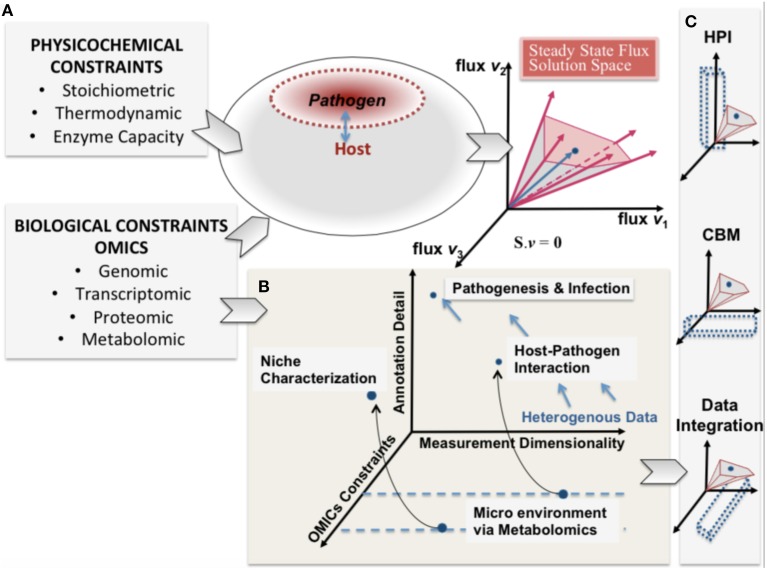
**A conceptual representation of integrating constraint-based modeling and omic data**. The heterogeneity of omic data (biological constraints) and their integration is represented in parallel with the phenotypic solution space of the high dimensional host-pathogen model derived from physicochemical constraints. The degree of constraints represented will depend on the measurement capability and also define a reference set of behaviors that are feasible. **(A)** enumerates the heterogeneity of constraints for both host and pathogen and the resultant mathematically feasible and the potential biologically relevant solution space. In **(B)** pathogenesis and infection are shown from the perspective of 3 dimensions (i) omics constraints (also determined by experimental constraints) (ii) Annotation detail (based on existing legacy data) and (iii) the measurement dimensionality (also defining dimensionality of data). **(C)** shows that understanding host-pathogen interaction would be possible at multiple scales by integrating heterogeneous data/measurements and constraint-based modeling algorithms. The opportunity afforded by the legacies of high throughput omics experimentation and systems-level mathematical models would help understand the emergent host-pathogen interaction.

Rudolph Virchow, a nineteenth century co-founder of pathology is credited with describing pathology as “physiology with obstacles” and specifying a “diseased state” as a quantitative deviation from normal function as a result of internal and external (i.e., environmental) influences (Virchow, 1958). Infections of a host by a pathogen can lead to acute and chronic pathological conditions. The process of infection by a pathogen can be viewed as a pathological process resulting from environmental stresses. These causal influences by the pathogen, onto the host, define the capabilities of the host and its pathogen can be expressed as constraints on the metabolic capabilities of the host and pathogen (Figure 1).

The continued development of high-throughput technologies are enabling profiling of multi-cellular and multi-organism environments (Gawronski et al., 2009; Han et al., 2010; Pacchiarotta et al., 2012; McAdam et al., 2014). Such advances enable the detailed measurement of molecular changes occurring in host-pathogen interactions (Kim and Weiss, 2008; Stavrinides et al., 2008; Beste et al., 2013; Le Chevalier et al., 2014; Schoen et al., 2014; Chang et al., 2015; Henningham et al., 2015; Yao and Rock, 2015). Generation of these large datasets, in the context of the complexity of pathogenesis, highlight the need for systems based approaches for integration into a cohesive biologically interpretable framework (Durmus et al., 2015). Constraint-based modeling is an ideal approach for a systematic, integrated analysis of these data. The approach is based on well-defined stoichiometric biochemical transformations (including mass balance, reaction capacity, and directionality) and gene-protein-reaction (GPR) relationships allow mapping and integration of multiple, disparate data types. These methods can incorporate heterogeneous data-types that represent all hierarchies in the reductionist causal chain of an organism, thus enabling prediction of emergent properties (Figure 1). Additionally, constraint-based models circumvent the problem of over fitting data, which often plagues strictly statistical based methods. There exist a number of freely available tutorials and implementation tools and packages enabling the use of reconstructions for modeling, analysis, and simulation in the literature (Schellenberger et al., 2011; Liao et al., 2012; Ebrahim et al., 2013; Sadhukhan and Raghunathan, 2014; Palsson, 2015).

## Where in the tree do host-pathogen models lie?

Constraint-based modeling in metabolism has its roots in microbial organisms, but has progressively grown in the past decades to describe complex multi-cellular organisms and various processes (Reed and Palsson, 2003; Mo et al., 2007; Feist et al., 2009; Karlsson et al., 2011; Osterlund et al., 2012). There has been a continual, systematic growth and progression of constraint-based models which initially began as the formulation of a core biochemical network as a linear optimization problem (Papoutsakis, 1984; Fell and Small, 1986; Varma et al., 1993). Further incorporation of additional layers of biological information through GPRs, thermodynamic constraints, and various high throughput data have increased the scope of the models beyond small species metabolism, to multi-cellular, multi-compartmental organisms (Duarte et al., 2007; Mo et al., 2007; Herrgård et al., 2008; Lewis et al., 2010; Ahn et al., 2011; Bordbar et al., 2011; Chang et al., 2011; Saha et al., 2011; Mintz-Oron et al., 2012; Seaver et al., 2012; Wang et al., 2012; Pornputtapong et al., 2015). This evolution in the field has been accompanied by a growth in associated methodologies (Lewis et al., 2012) and new discoveries (Ellis et al., 2009; Ahn et al., 2011; Frezza et al., 2011; Thomas et al., 2014; Väremo et al., 2015). The importance of metabolism in understanding the process of infections and host pathogen relationships is increasingly being recognized (Han et al., 2010; Kafsack and Llinás, 2010; Pacchiarotta et al., 2012; Beste et al., 2013; Mcconville, 2014; Schoen et al., 2014; Yao and Rock, 2015). The cellular environment and repertoire of available metabolites is critical in characterizing and understanding how a pathogen interacts with and infects the host and constraint-based approaches can provide value insight into mechanisms of resistance and potentially new drug treatment targets (Chavali et al., 2008; Huthmacher et al., 2010; Bazzani et al., 2012; Kim et al., 2013; Shoaie and Nielsen, 2014; Tymoshenko et al., 2015).

In the “evolutionary tree” of constraint-based models, host-pathogen models lie between multi-cellular models, pathogen modeling, and new constraints/data integration approaches. There are now numerous exciting frontiers in the growth of these models, including the scope, incorporation of physicochemical constraints, multi-tissue, and multi-organism models (Cakir et al., 2006; Kümmel et al., 2006a,b; Beg et al., 2007; Duarte et al., 2007; Mo et al., 2007; Herrgård et al., 2008; Lewis et al., 2010; Ahn et al., 2011; Bordbar et al., 2011; Chang et al., 2011; Saha et al., 2011; Metris et al., 2012; Mintz-Oron et al., 2012; Seaver et al., 2012; Wang et al., 2012; Pornputtapong et al., 2015). Some of the challenges regarding model integration will be shared with related areas of multi-cellular constraint-based modeling, such as modeling microbial communities (Stolyar et al., 2007; Karlsson et al., 2011; Shoaie and Nielsen, 2014) and the development of new methods characterizing the interaction between cellular interactions between different species (Harcombe et al., 2014). Notable differences between host pathogen modeling and microbial community modeling include the specification of cellular objectives and constraints as well as differences in spatial compartmentalization (microbial community modeling will generally involve interaction through a shared extracellular space, whereas host pathogen models may interact through additional compartments; see below). We confine the scope of this work to focus on host-pathogen constraint-based modeling that entails the explicit integration of two genome-scale (or cell scale) constraint-based models. The purpose of this article is to describe a systematic methodology leading to successful integration of constraint-based host-pathogen models. Although there have been a relatively small number of actual host-pathogen (hp) models reconstructed to date, the existing studies have produced interesting results and have taken steps toward elucidating the pathway forward for future investigations (Raghunathan et al., 2009, 2010; Bordbar et al., 2010; Sadhukhan and Raghunathan, 2014).

The extracellular environment has an influential effect on the phenotype state and behavior of cells, thus pathogens have different biochemical phenotypes when inside the host versus outside the host and that the host cells will be affected in some manner by the pathogen and vice-versa. Many current experimental techniques enable characterization of these different states (Deatherage Kaiser et al., 2013). The generation of such data results in the technical challenge of simultaneous interpretation and analysis of genomic, proteomic, and/or metabolomics data of two independent, yet interacting organisms. The ability to derive meaningful interpretations of such data requires a computational setting which enables mapping and integrating data in a coherent format that further allows the data to be analyzed simultaneously, beyond simply looking at correlations or fitting presumed associations to an expected model. The constraint-based modeling framework affords a means to do so.

**Figure 2:**
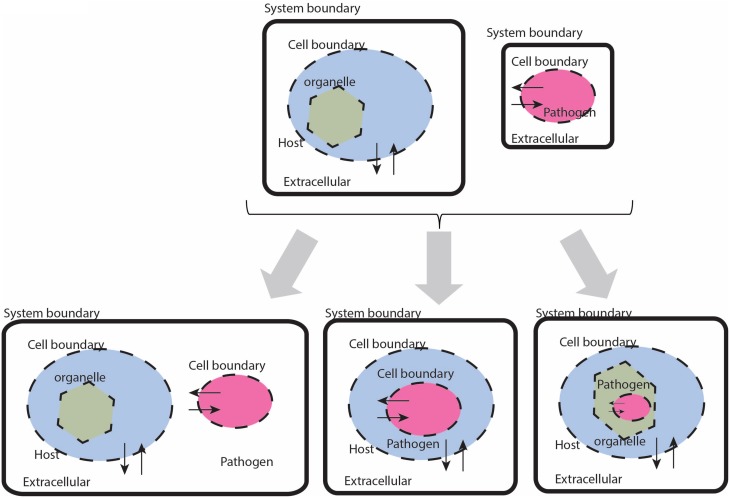
**Cartoon based schematic representation of different types of interactions between a host and pathogen model, with special attention to system boundaries**. The system boundary is clearly delineated with a solid black line, whereas organism boundaries are dashed lines (the organelle boundary is represented with a thinner black line. Note that with this formulation, individual models will be required to have exchange reactions for every metabolite that has a trans-membrane transporter.

While there are a seemingly innumerable number of ways that pathogens have evolved to infect and reside their chosen host tissues and organs, in general terms there are few places these organisms can localize: intracellular, extracellular—interstitial, extracellular—intravascular, extracellular—transcellular, and “semi-open” spaces (e.g., the respiratory or alimentary tracts, etc.). In the constraint-based framework, there are three types of compartment based interactions between the host and pathogen (defined by the interaction boundary as defined by the pathogen's cell wall): extracellular, intracellular:cytosolic, intracellular:intra-organelle (Figure 2). Within the intracellular environment, there are multiple compartments that a pathogen may localize and life cycles of pathogens in some organisms reside in different compartments, depending on the stage of infection. These details are organism specific and are addressed on a case-by-case basis.

## Reconstructing a host-pathogen constraint-based model

### Formulation of a description of a biochemical network as a constraint-based optimization problem

The formulation of metabolic network descriptions in terms of constraint-based modeling and relation optimization methods is rooted in applying the principle of mass conservation and thermodynamic constraints to these networks and has previously been described in detail (Fell and Small, 1986; Varma et al., 1993; Orth et al., 2010; Palsson, 2015). Integration of host-pathogen models requires two curated stoichiometric representations of metabolic networks, for which the minimum requirements are a stoichiometric matrix and a flux vector with upper and lower bounds,

(1a) $${\text{S}}_{\text{h}}\;\cdot \;{\text{v}}_{\text{h}}=0$$

(1b) $${\text{v}}_{\text{h}}^{\text{lb}}\leq {\text{v}}_{\text{h}}\leq v_{\text{h}}^{\text{ub}}$$

for the host and,

(2a) $${\text{S}}_{\text{p}}\;\cdot \;{\text{v}}_{\text{p}}=0$$

(2b) $${\text{v}}_{\text{p}}^{\text{lb}}\leq {\text{v}}_{\text{p}}\leq {\text{v}}_{\text{p}}^{\text{ub}}$$

for the pathogen, with S_h_ ∈ R^mh x nh^, S_p_ ∈ R^mp x np^, v_h_ ∈ R^nh^, and v_p_ ∈ R^np^ (see Notations/Abbreviations).

For host-pathogen modeling, Equations (1) and (2) are not applied under the strict steady state assumption, but rather along the lines of a quasi-homeostatic state for which we enforce mass conservation over a time scale of interest. With this consideration in mind, the calculation of interest is rarely a specific flux point, but rather a group of points reflecting a particular flux state (or a region within the right null space) corresponding to a particular phenotype that can be differentiated from other qualitatively different flux states. Identification of such regions often may not require the specification of a metabolic objective function, in which case non-objective based methods, such as sampling, may be appropriate (Savinell and Palsson, 1992; Barrett et al., 2006; Schellenberger and Palsson, 2009; Bordel et al., 2010).

Pre-existing curated, functional models are a necessary but not sufficient requirement for building an hp model. Even if two models are well posed, integration of the two may result in discrepancies as a result of multiple factors including,

- Error ranges in experimentally derived values (such as biomass components).
- Incorporation of data from different experimental conditions that may not be consistent with one another from a mass balance or thermodynamic perspective.
- Limitations in biological scope of each respective model.
- Lack of knowledge about the true or underlying biological objectives.

**Figure 3:**
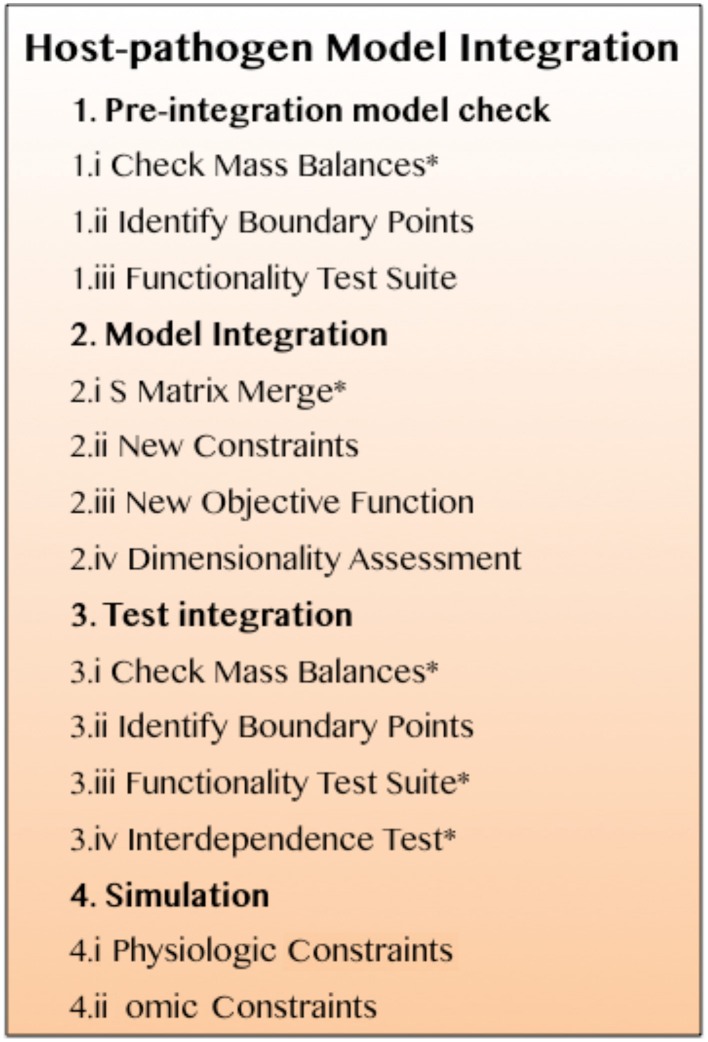
**A systematic procedure for successful, functional integration of a constraint-based host-pathogen model**. Details are described in the main text. The asterisks identify steps that require iterative revisions if the models fail the corresponding test (see ^*^Iteration/revision checkpoints in the main text).

Additional, important considerations to be made when transitioning from the analysis of an isolated pathogen to a host-pathogen model include, simulating different conditions with different data sets, simulating the same species under different states versus different species under similar conditions, and specification of the conditions in which gene lethality knockout/knockdown studies or drug sensitivity screens are performed and their applicability to host-pathogen infectious states. These issues highlight the need for a systematic methodology for integrating host-pathogen models. Constraint-based host-pathogen modeling can be viewed as a generalizable, systematic, multi-tiered process with iterative sub-steps (Figure 3). Each step includes multiple sub-steps that require simulations or calculations to be performed, often in an iterative fashion. A systematic approach for building and testing the models during the integration process will help make the debugging process more transparent and the more directed identification of potential problems.

#### Step 1. pre-integration model check

This initial step serves as a “sanity check” to avoid problems during the subsequent integration components of the study. Although current standards for building curated network reconstructions generally require critical quality control/quality assurance steps to avoid spurious behavior from ill-posed models, prior to integration, there are a number of tests that must be completed for each model to confirm the models have been constructed and formulated appropriately.

**Figure 4:**
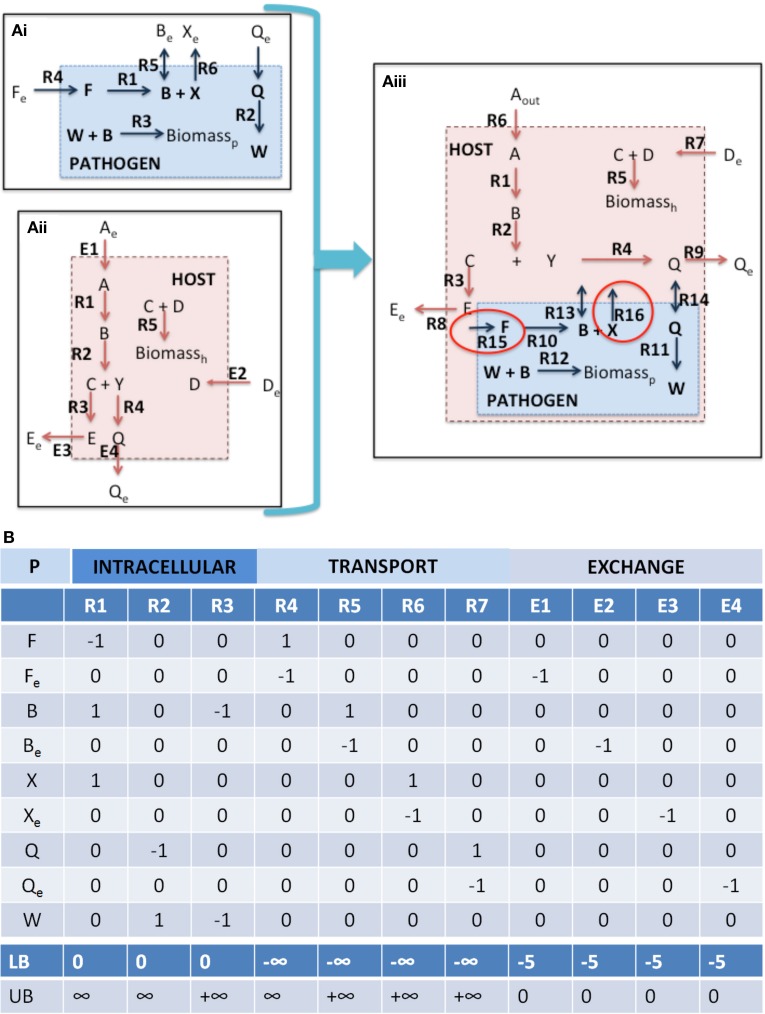

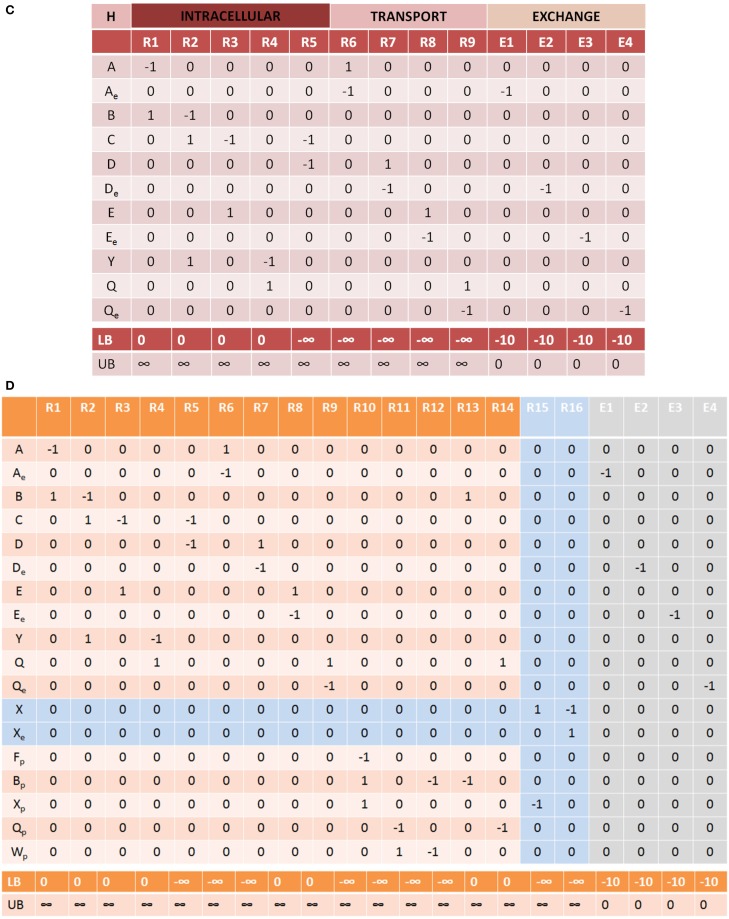
**Integration of toy a host cell model with an intracellular pathogen model. (A)** depicts a cartoon schematic of a pathogen model, host model, and integrated host-pathogen model with the corresponding stoichiometric matrices for each of the models (**B** corresponds to **Ai**, **C** corresponds to **Aii**, and **D** depicts the stoichiometric matrix for the hp network in **Aiii**). Note that when the pathogen “infects” the host the transporters for metabolites B and Q enable usurpation of host resources and will consequently limit the biomass construction capabilities of the host (potentially the pathogen as well, depending on the size of the demand). In the provided example, metabolites F and X are not within the intracellular environment of the host, thus R10, R15, and R16 will not be able to carry a flux. In spite of this however, since there is a transporter for metabolite B, the pathogen biomass can still be produced even though R10 will not be able to carry a flux. It is also possible that metabolite F and/or X actually *are* available in the host, but that the particular metabolites were outside the scope of the reconstruction at that time. In this case, the host model can be updated to include the relevant reactions that would make the metabolites available within the intracellular environment. The multiple points within the protocol that would allow for evaluation of the appropriateness of including additional reactions during the iterative revisions, particularly Steps 3.iii, 3.iv, and 4.i. Intracellular organelles are not described in this toy example, however if the pathogen infects the host and resides within a particular organelle within the host cell, the procedure would be the same. Note that the exchange reactions are not explicitly illustrated within the figures, but the columns are present in the stoichiometric matrices.

*1.i Check mass balances (“No free lunch”)*. Well curated models should be free of errors that may lead to violation of mass conservations constraints. However prior to integration, each model should be tested to confirm this, i.e., all uptake exchange reactions should be closed and flux variability analysis (FVA) (Mahadevan and Schilling, 2003) should be performed on the entire model, in order to confirm that there is no *net* production of *any* metabolite, when no substrates are available for uptake. In the toy model depicted in Figure 4, it is clear that if the substrates for the host and pathogen are not available (F_e_, A_e_, and D_e_), then none of the secreted compounds (B_e_, X_e_, E_e_, Q_e_) can be produced.
*1.ii Identify boundary points*. The simplest approach for identification of the boundary points for a model is through FVA. Although this step can technically be included in the Functionality Test Suite, FVA is such a useful tool for debugging and initial assessment of models, that it is judicious to include this as a mandatory step in the model integration protocol. Under general uptake conditions (that are still biologically and thermodynamically feasible), FVA is performed with subsequent calculation of the flux spans. This assessment will enable the determination of the ranges of all reactions and the potential identification of “closed” reactions, any unbounded reactions, etc.
*1.iii Functionality test suite*. Prior to integration there should be a pre-defined set of simulation condition(s) and reaction optimizations in order to test and confirm desired functionality of the model (Duarte et al., 2007); this set of reactions comprise the Functionality Test Suite (FTS). The FTS can contain any number of desired tests and simulations to ensure appropriate physiologic behavior of the model, examples include biomass production under different growth conditions, specific gene knockout lethality experiments, inability to growth under specified conditions, or any other appropriate test that would evaluate the physiological/biological characterization of the model or the underlying mathematical definition.

#### Step 2. model integration

Although stoichiometric matrix integration of two models is trivial from a technical standpoint, the functional integration of a simulation-capable host and pathogen network reconstruction is a non-trivial process. The panels in Figure 4 provide a concrete illustration of the integration of two toy models.

**Figure 5:**
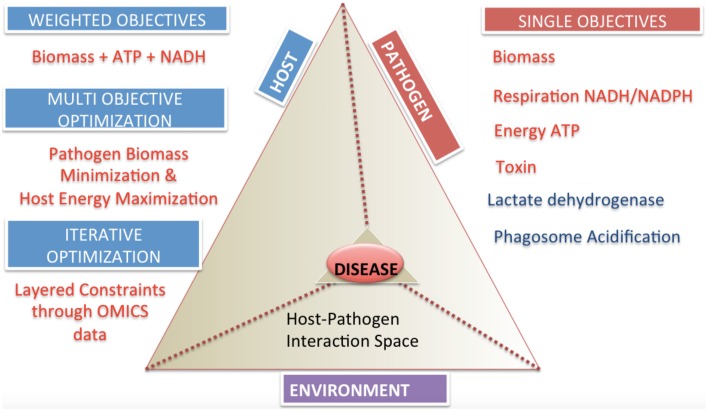
**Categories and classifications of objective functions in host-pathogen models**. The host-pathogen interaction pyramid is shown that integrates host, pathogen and environment to result in the diseased state phenotype. The diseased state can be queried with the correct formulation of objective functions as discussed for the three components delineated here. The two sides of the triangle represent the host and the pathogen and the connecting side represents the environment or niche. The sides converge on the vertex of the prism reflecting the lethal disease state. The space outside the host pathogen interaction prism lists objectives and their classification. Single objectives help define pathogen or host state, while multi-objectives or weighted objective functions allow definition of complex phenotypes.

*2.i S matrix merge*. The stoichiometric matrices are joined through a compartment specific, row wise-merge (Figure 4). Generally compartment specific reactions (i.e., the compartment in which nutrients are directly exchanged between the host and pathogen) will not be shared between the host and pathogen model, however it is important to confirm this when constructing the new stoichiometric matrix.
  (3) $${\text{m}}_{\text{hp}}<{\text{m}}_{\text{h}}{\text{+m}}_{\text{p}}$$
  (4) $${\text{n}}_{\text{hp}}\approx {\text{n}}_{\text{h}}{\text{+n}}_{\text{p}}$$
  Note that Equation 3 is defined by an inequality, whereas Equation (4) is an approximation. The degree of integration and subsequent complexity of the interactions between the models is dependent on the number of metabolites that overlap between the two organisms. If the organisms do not share any metabolites (m_hp_ = m_h_ + m_p_), then integration of the two models will not result in any novel predictions. On the other hand, the number of reactions in the combined network may be equal to, less than, or greater than the sum of the two individual models. In toy model integration depicted in Figure 4, m_p_ = 9, m_h_ = 11, and m_hp_ = 18, satisfying the Equation (3) inequality. For the toy model, Equation (4) is an equality, since the number of reactions in the combined model is equal to the sum of the individual models.
*2.ii New constraints*. Integration of two models includes the introduction of additional constraints that will make the simulation environment context specific and more representative of the actual biological environment.
  - Nutrient availability and demand. These constraints are the most simple to implement and should provide strong coupling between the host and pathogen. In addition to biomass (growth and non-growth associated constraints), additional condition dependent constraints can be incorporated, for example demands on micronutrients, sequestration of metabolites, etc. (Rodriguez et al., 2002; Pan et al., 2010; Weiss and Schaible, 2015). For example in the toy model (Figure 4), further curation may be needed in order to identify the appropriate bounds for the intracellular pathogen uptake conditions as well as any potential new demands on available host nutrients (not depicted in this example).
  - Coupling constraints. The host and pathogen networks will interact by virtue of the compartment specific shared metabolites. However, physiologically, the infection of a host by the pathogen frequently results in additional interdependencies between the two species, such as competition for a shared resource. Coupling constraints are the mathematical relationships formalizing the explicitly link between the host and pathogen models together as a constraint. This relationship may take the form as an interaction between two molecules, concordant activity between two enzymes, or some other biological process. For example,
    (5) $${\text{v}}_{\text{i}}^{\text{h}}{+/-\text{v}}_{\text{j}}^{\text{p}}=\alpha_{\text{k}}$$
    in which α_k_ is a physiologic constant or data dependent variable (e.g., protein production rates, mRNA expression, etc.). Non-unity coefficients can be added to the reactions, if there is known to be a fixed, stoichiometric balance between the two (or more) reactions. Depending on the type of relationship represented, this relationship can be expressed as a continuous flux based problem, or a discontinuous/discrete problem; the latter would require formulation as a Mixed Integer Linear Programming (MILP) problem (Burgard et al., 2001; Phalakornkule et al., 2001; Pharkya et al., 2004; Kumar and Maranas, 2009). In the case of hp models, MILP constraints may be used to express conditionally active reaction constraints. For example in the toy model depicted in Figure 4, if pathogen growth (i.e., biomass production, Figure 4Aiii, R12) were to only occur if the host cell would take up a particular metabolite (e.g., metabolite D, Figure 4Aiii, R7).
  - State changes. To date methodologies for representing changes in infectious states during an infectious cycle or a pathogens life cycle have been represented as discrete, independent simulations. Depending on the type of data that is available, context specific models can be constructed for each different state or alternatively, conditional, state dependent constraints MILP constraints can be defined.
*2.iii New objectives*. Flux balance analysis is an optimization problem and while there are formulations of the constraint-based modeling problem that do not require the definition of a metabolic objective to be optimized (Lewis et al., 2012), the incorporation of an objective function to be maximized or minimized is often of great utility, since it enables more specific predictions to be made by reducing the size of the steady state solution space (right null space). The definition and identification of objective functions is an area of great importance in these models (particularly mammalian cell models) that is a very rich area for exploration and in need of further development in the current literature (Khannapho et al., 2008; Schuetz et al., 2012; Shoval et al., 2012; Szekely et al., 2013). The flexibility in designing cellular objectives to tailor hp specific responses is critical for achieving success with this approach. The biomass objective function has been discussed in great detail and is generally considered in terms of two general components: a growth associated component (accounting for biomass constituent components) and a non-growth associated component (Feist and Palsson, 2010). The biomass reaction can be treated as a constraint on the system or as a prediction to be made by the model as a means to validate a network reconstruction (Price et al., 2004). Since the growth of the solution space can increase dramatically when two models are merged, defining lower bound constraints on growth associated and non-growth associated biomass functions for the host or pathogen is a practical necessity in order to calculate meaningful results. Organism specific objectives may be developed from the new constraints that are defined or identified experimentally.
  The specification of appropriate objective functions requires detailed understanding of pathogen physiology and host pathogen interactions. These can be separated into two general categories, single objective and multi-objective problems (Figure 5). Examples of potential objective functions include but are not restricted to, the (pathogen) biomass pseudo-reaction, iron acquisition (Ratledge and Dover, 2000; Nairz et al., 2015), lactate dehydrogenase levels as a indicator level of cytotoxicity (Korzeniewski and Callewaert, 1983; Decker and Lohmann-Matthes, 1988), enterotoxin production, pathogen specific metabolite production (Glickman et al., 2000; Takayama et al., 2005), reactive oxygen species minimization (Brynildsen et al., 2013), and other critical minerals and metabolites.
  Multi-objective functions are more complex, but may reflect a more accurate representation of the biology (Gianchandani et al., 2008; Schuetz et al., 2012; Zakrzewski et al., 2012). The practical challenge is knowledge of the adequate data to specify these objectives.
  - Weighted objectives. New objective functions can be constructed from the linear combination of reactions representing cellular demands and requirements. By combining different reactions together to generate “compound” or weighted objectives, more complex behavior can be captured. The obvious weakness of this approach is that the stoichiometric coefficients are fixed for the different components, thus this approach is only applicable in situations in which fluxes (or metabolite production/consumption) occur in fixed ratios with one another (as in biomass).
  - Bi-level optimizations across host-pathogen boundaries. Bi-level optimization algorithms designed for bioengineering and evolutionary objectives (Burgard et al., 2003; Zomorrodi and Maranas, 2012) can be extended and applied to understand the dynamics across host and pathogen during an interaction. Depending on the experimental conditions, this may include optimization of pathogen biomass within the host. For example there may be competing objective functions, as in the case of maximization of pathogen biomass and host biomass concurrently or in diametric opposition, i.e., maximization of pathogen biomass with minimization of host substrate availability (either through minimization of pathogen transport uptake or host transport uptake).
  - Multi-level optimization. Although, computationally intensive, multi-objective optimization (Zakrzewski et al., 2012; Zomorrodi et al., 2014) can enable a more accurate representation and in turn more accurate mathematical simulation of the host-pathogen interaction.
  - Step wise algorithmic multi-objectives i.e., sequential optimizations that apply additional constraints at each iteration. Iterative optimizations are approach for including multilayered omic or physiological constraints allow to be added in order to asses hp behavior in varying environments or host niche's (D'Huys et al., 2012). Such approaches also support the integration of heterogeneous data types. A limitation of this approach is that the optimization is order dependent, and thus may be a more valuable tool for assessing the effects of different constraints as opposed to a more physiological objective.
*2.iv Dimensionality assessment*. Dimensionality assessment of the network includes determining the size of the network, including the number of metabolites and reactions, as well as the size of the “functional” space of the network, such as the right and left null spaces. These components can be directly calculated from m, n, and the rank of the new stoichiometric matrix S_hp_. These quantities can be used to calculate the size of the right and left null spaces (N_r_ = n–R and N_l_ = m–R). These simple calculations allow assessment of the dimensionality of the new model (in terms of number of components and reactions, as well as the steady state solution space), which will assist in debugging and interpretation of calculated results and simulations (notably Steps 3 and 4). Table 1 summarizes these results for the toy models described in Figure 4. Knowledge of the right null space in particular is useful when debugging potential problems and interpreting simulation results. Integration of the two models results in an increase in the steady state solution space (i.e., at least 1 new independent metabolic pathway) as a result of the integration from the host and pathogen. The left null space contains the conserved chemical moieties within a network (Famili and Palsson, 2003; Sauro and Ingalls, 2004). The size and contents of the left null space can be used to understand how metabolites may pool together based on network structure and often provides functional insights (Famili and Palsson, 2003; Thomas et al., 2014).
  Additional graph theoretic measures can be calculated (Girvan and Newman, 2002; Estrada and Rodríguez-Velázquez, 2005; Fatumo et al., 2011), although their utility in assessment of functional characteristics and trouble-shooting in the context of hp model construction is currently limited.

**Table 1:** **Descriptive summary of the toy models**.

	**Pathogen**	**Host**	**Host-pathogen**
Number of metabolites	9	11	18
Number of reactions	11	13	20
Right null space dimension	2	2	3
Left null space dimension	0	0	1
Rank	9	11	17
Mean betweenness centrality	0.11	2.64	4.33

The sizes of the stoichiometric matrices and the respective right and left null spaces. The steady state flux states reside in the right null space (calculated from applying mass conservation constraints). The left null space size describes the number of “conserved” metabolic moieties. In the case of the toy model, the left null space compound is the metabolite that cannot be imported into the pathogen, because the host model does not import or metabolize it.

#### Step 3. integrated host-pathogen testing

On the surface, integration of two models is a trivial step given the general simplicity of the basic formulation of constraint-based models. The initial technical challenge is to identify the overlapping set of metabolites and corresponding abbreviation mappings between the host and pathogen metabolites.

Although there are laudable efforts to use standardized nomenclature (Radrich et al., 2010; Dräger and Palsson, 2014), a persistent challenge in the field is the use of different abbreviations and nomenclature, which has often required dedicated efforts to reconcile multiple versions of network reconstructions (Herrgård et al., 2008; Thiele and Palsson, 2010). Fortunately, however, for host pathogen models, every metabolite within the two models does not need to be compared, but rather just the boundary metabolites, which are generally a fraction of the total number of metabolites in a model. This is relatively straightforward through the comparison of abbreviations, if the reconstruction has been appropriately annotated [e.g., molecular formula, SMILES (Weininger, 1988), ChEBI (Degtyarenko et al., 2008), etc.]. Once the shared metabolite complement is identified, the stoichiometric matrices can be merged (Figure 4). However, “blind” integration without proper quality control/quality assurance and test conditions in place, the results will be difficult and quickly overwhelming to interpret.

The first three sub-steps for Step 3 are similar to Step 1. Depending on the type and complexity of new constraints that are applied to the integrated host-pathogen model, there are situations that may introduce behavior that violates mass conservation, thus it is necessary to confirm that no “free metabolites” are produced. For situations in which the pathogen is an intracellular organism, the test needs to be applied to the host-pathogen model, as well as the isolated pathogen, within the host.

*3.i Check mass balances*. See Step 2. Model Integration, 2.i and Figure 3, 2.i.
*3.ii Identify boundary points*. Identification of the right null space boundary points through FVA of the host-pathogen draft model will permit a detailed, yet global view of the capabilities of the combined host-pathogen and enable comparisons to the individual organisms (Step 1.ii). This comparison may identify reactions or constraints that may require revisions to be made. For example, upper bounds constraints may need to be increased if the combined model enables the pathogen to exceed the upper limit of some reactions in comparison to the isolated organism. In the case of the toy model illustrated in Figure 4 (integrated host pathogen model), if host's intracellular environment is much richer than the “open” environment for the pathogen and in the infected state, R4 >> R12 (Figure 4Aiii), then the upper bound of R12 may need be increased in order to permit a larger potential rate of biomass accumulation.
*3.iii The functionality test suite*. The functionality test suite of the combined host-pathogen model will also enable a basis for comparison with 1.iii and assist subsequent analyses (Step 4). Note that the FTS for the individual host and pathogen models may not be identical to the hp set of test reactions, since the metabolic network capabilities of the host and pathogen will not be identical in the infected versus uninfected states.
*3.iv Interdependence test* This test requires identifying objective functions that are expected to influence or be influenced by the coupling between the host and pathogen. The biomass function is a very good candidate for such tests, as it is connected to many different pathways within each respective organism, and subsequently more likely to be directly connected to the host (or pathogen). The biomass pseudo-reaction, however, is not the only possible objective to test and other cellular/metabolic functions may be of utility, such as ATP production, oxidative phosphorylation, or constraints on secretion/uptake of particular metabolites (Schuetz et al., 2007, 2012; Khannapho et al., 2008; García Saánchez and Torres Sáez, 2014).
  The interdependence test involves two steps,
  - Calculate the optimal host biomass production in the host-pathogen model, then fix the lower bound of the host biomass reaction to a specified value (1-ε_1_) and then optimize for the biomass of the pathogen:
    $$\begin{array}{l} \text{For }\alpha_{\text{1}}=\text{max}({\text{v}}_{\text{hp}}^{\text{BM},\text{h}}),\\ \text{set}:\text{ lower bound}({\text{v}}_{\text{hp}}^{\text{BM},\text{h}})\geq (\text{1}-\varepsilon_{\text{1}})\alpha_{\text{1}}\\ \text{max }({\text{v}}_{\text{hp}}^{\text{BM},\text{p}})=\beta_{\text{2}} \end{array}$$
  - Calculate the optimal pathogen biomass production in the host-pathogen model, then fix the lower bound of the pathogen biomass reaction to a specified value (1-ε_2_) and then optimize for the biomass of the host:
    $$\begin{array}{l} \text{For }\beta_{\text{1}}=\text{max}({\text{v}}_{\text{hp}}^{\text{BM},\text{p}}),\\ \text{set}:\text{ lower bound}({\text{v}}_{\text{hp}}^{\text{BM},\text{h}})\geq (\text{1}-\varepsilon_{\text{2}})\beta_{\text{1}}\\ \text{max }({\text{v}}_{\text{hp}}^{\text{BM},\text{h}})=\alpha_{\text{2}} \end{array}$$
    Comparison of α_1_ to α_2_ as well as β_1_ to β_2_ provides an indication of the degree of coupling between the two models. If α_1_ ≈ α_2_ and β_1_ ≈ β_2_, then there is no significant coupling between the two models. Conversely, if these values are significantly different from one another then there is evidence of interaction between the models on a metabolic level. It is more common to have uni-directional coupling between the models, often in favor of the pathogen, i.e., β_1_ ≈ β_2_ and α_1_ > α_2_ due to usurpation of host resources by the pathogen. The ε coefficients are empiric, simulation based parameter whose value will vary depending on the specific organism, the biomass composition, and the media growth conditions. The “ideal” ε will be large enough to force the consumption of metabolites and resources required to produce biomass, but small enough not to introduce a significant bias in the flux state. When the coefficient ε is equal to 0, then the interdependence test is equivalent to a stepwise optimization comparison. Generally the coefficient ε is small, typically 0.01–0.1, when the biomass function is used. A phase portrait analysis (Edwards et al., 2002) may be useful in assessing and determining an appropriate ε value. Since ε is a specified value, the degree of coupling between the host and pathogen can be titrated to a certain degree. Note that since the growth rates of the host and pathogen may be very different from one another, then ε_1_ and ε_2_ may be different from one another.
    Since the corners of the right null space generally become increasingly acute as the size of the model increases, when the biomass is fixed at the optimum level there is a dramatic decrease in the available alternative solutions. However when this constraint is relaxed even by a small amount, the number of alternative solution points dramatically expands; thus in order to assess robust coupling between the host and pathogen, generally a non-zero ε should be chosen.
    For example in the toy model depicted in Figure 4, the pathogen biomass function is dependent on substrates provided by the host. If the uptake of metabolite A (Figure 4Aiii, R6) is unbounded (or not known to have any constraint), then the intra-cellular reproduction of the pathogen is not significantly constrained and independent of the host. However, if the host's uptake of metabolite A is limited, then the pathogen's growth rate will be limited. A common source of error and potential difficulty during the integration of a host and pathogen model is for the pathogen biomass production rate lower bound to be set above the availability of the particular metabolite (i.e., either the host uptake constraints or the host to pathogen transport reactions), which results in a non-functional model. In these cases, the data used for defining the constraints must be re-evaluated and either the constraints would need to be revised or there additional reactions would need to be added to provide alternative routes for availability of the requisite metabolite(s).

#### Step 4. simulation

The type of simulation of interest is principally dependent on (1) the type of data available, (2) the biological organism of interest, and (3) the data available to validate or test the simulations. Due to the broad scope and scale of the realm of possible simulations, it is not practical to specify a list of calculations that can be applied for every condition. The purpose of this step is to assist in bridging the construction of the model to a meaningful use of the model in the subsequent analysis steps. A common characteristic of the simulation stage however involves evaluation steps and the question of how to reconcile inconsistent results between the model simulations and experimental observations. Suffice it to say that the use of integrated omic data is one of the most successful aspects of constraint-based modeling and there are a number of growing methods being developed for incorporating genomic sequence, transcriptomic, proteomic, and metabolomic data; interested readers are referred to available review articles outlining some of these methods (Blazier and Papin, 2012; Lewis et al., 2012; Wang et al., 2012; Machado and Herrgård, 2014; Robaina Estévez and Nikoloski, 2014). For the purpose of organization and simplifying the debugging process, the simulation tests can be classified into two general areas,

*4.i Physiological constraints*. Simulations validating (or invalidating) predictions of the model using available physiologic data sets.
*4.ii Omic constraints*. Simulations validating (or invalidating) predictions of the hp model through omic data sets.

#### ^*^Iteration/revision checkpoints

“Failure” of specific steps in the protocol (Figure 3) requires an iterative adjustment to be made through revision of the original models, the integration step, or in some cases further literature curation and updating of model content or constraints.

*1.i Check mass balances (individual models)*. Failure: Return to Step 1 (or before). If either the host or the pathogen model result in violation of mass conservation constraints, then the respective model needs to be critically evaluated and debugged, so that the offending reaction(s) is/are identified and removed or adjusted appropriately. The appropriate definition and representation of system boundaries is a simple, yet critical step. Consequences of undefined or inappropriately defined system boundaries will lead to an ill-formulated model that will likely result in mass balance errors. The cartoon illustration in Figure 2 highlights the appropriate definition of system boundaries when before and after integration of a host with a pathogen reconstruction. The most direct and common consequence of poorly defined boundaries is an ill-formulated description of the optimization problem with subsequent errors in mass balance, resulting in irrelevant and even non-sensical results.

##### Dimensionality assessment

Failure: Return to Step 2.i. “Failure” of this step constitutes violation of Equation (3). When merging two (or more reconstructions) there must be a mapping between metabolites that are shared by each of the two models. At minimum there must be at least 1 metabolite that is shared between each model, although in practice there are generally at least 30–40 metabolites that are shared. Once compartment specific identification of shared metabolites has been performed, then the two sets of models can be merged through merging the stoichiometric matrices “row-wise.” If m_h_ + m_p_ = m_hp_, then there has likely been an error in integration [either through formulation of the problem (Step 1) or implementation of the matrix merge (Step 2.i)]. As noted above, in general, n_hp_ ≈ n_h_ + n_p_, with the approximation being dependent on whether additional constraints or new objective reactions are added in the integrated network.

##### Check mass balances (host-pathogen model)

Failure: Return to 2.i. If the integrated host-pathogen model results in violation of mass conservation, but the individual models did not, then there was an error in the model integration (Steps 2.i-2.iii). Evaluation of the boundary exchanges of the pathogen should be the first area of critical evaluation.

##### Functionality test suite

Failure: Return to 1.iii. Depending on the type of error and the type of functional test, this may be “real” or it may reflect incomplete knowledge (such as an incompletely defined biomass function). Failures in the FTS should be analyzed to determine the source of the limited constraint (the FVA calculations 3.ii can be helpful in tracking this within the network). Once the cause of the failure is identified, it needs to be determined if this is the result of erroneous reaction constraints or a real prediction (i.e., a reaction that is active in the “uninfected” state but is inactive in the infected state). Referral to the primary literature is frequently needed to resolve these issues.

##### Interdependence test

Failure: Return to Step 1 and 2.ii. The lack of interdependence may require revision of the model(s) (through additional curation and scope expansion) and/or re-assessment of the new constraints and objective functions that were added. For example, in the toy model depicted in Figure 4Aiii, further evaluation of the literature may suggest that R15 and/or R16 are active in the pathogen during infections, which would require further evaluation as to how metabolites F and/or X, respectively are made available to the pathogen inside the host cell.

##### Simulation

Inconsistencies between model predictions and observed experimental results or invalidating predictions should first be assessed in terms of the model and how the specific prediction was made, i.e., identification of the specific pathways leading to the calculated results. If there is no evidence to suggest a model related or numerical error, then there will need to be further perusal of the literature. For example in Figure 4Aiii, if there is biochemical or physiologic evidence in the literature suggesting that biochemical transformation carried out by R10 should be active (and able to carry a flux) in the infected state, then there needs to be further evaluation of the literature to determine how metabolite F is taken into the cell, or if there exists an alternative pathway for production of metabolite F within the pathogen (or host). This example also highlights the need for multiple iterative steps that often necessitate re-evaluation of the primary literature. In this case, the pathogen is still able to grow within the host, so there were no errors in Steps 3.i, 3.ii, 3.iii, or 3.iv (assuming that R10 was not contained in the FTS). This example is also illustrative of the need for the multiple checkpoints in the protocol (Figure 3) and the necessity of re-evaluating results and possibly revising the model(s) at each step of the integration process.

## Current state of the art and future outlook

The systematic procedure described above enables construction of host-pathogen constraint-based models that is applicable to organisms ranging from obligate parasites to multi-cellular pathogens, including viruses, bacteria, and fungi. The methods described above are most directly relevant and applicable to bacterial and fungal organisms. Viruses and parasitic organisms each demonstrate characteristics that may require further considerations, particularly with respect to conditional (e.g., transcription) dependent constraints. Some parasites are multi-cellular organisms that are capable of residing in multiple tissues within a host, thus the challenge by some of these organisms will require the integration of multiple, multi-cellular models. This process will be more involved, but will include the same systematic process. One should recognize the importance of “buffering” compartments and should include them, as they may play an important role in balancing protons, water, phosphate, etc.

Achievement of the steps outlined in Figure 3 will result in a functional host-pathogen model that should represent a more biologically accurate, quantitative, simulatable description of the interaction between a host and pathogen (Figure 5), in turn enabling a more objective, quantitative assessment of the interactions between these cells. Interrogation of these hp models would allow probing pathogen adaptation and carbon source utilization *in vivo* and host manipulation by pathogen. Such models should then be used to answer questions regarding causality during the infection process, condition dependent (or context specific) differences, and ultimately advance diagnosis and treatment related challenges by providing an environment to evaluate and generate hypothesis as well as interpret and analyze data.

The ability to measure and represent data on a genome-scale and the development of constraints based modeling strategies can help explore the complex host-pathogen interaction space (Figure 5). While the methods have reached a degree of maturity that enable the application to a wide range of conditions, there still remain many areas that deserve further exploration, including more elegant representation of changes in the environment (e.g., pH changes between different compartments and the associated charge changes that may occur with certain species) as well as more fluid descriptions in the transitions between different growth stages (e.g., rather than static representations for each stage, developing the analog of kinetic models, in which the change from one state to another can be simulated).

The process of host infection is complex and future developments will build upon studies that have, for example, investigated immune responsive signaling pathways such as the Toll-like receptor (Li et al., 2009) as well as the dynamics of pathogen metabolism (Penkler et al., 2015). With continual developments in approaches to expand the scope of reconstructions (Thiele et al., 2009; Lerman et al., 2012) and the development of new methods and approaches for generating genome scale network reconstructions (Overbeek et al., 2005; Henry et al., 2010; Monk et al., 2013), it is anticipated that there will be a dramatic rise in the development of hp models. Ultimately the objective of integrative constraint-based methods is to develop new strategies for treatment of pathogenic infections through novel target identification and new combination therapies for treatment (Trawick and Schilling, 2006; Jamshidi and Palsson, 2007; Karlsson et al., 2011; Chavali et al., 2012).

Constraint-based modeling allows meeting the challenge of complex omic data integration across time and space at multiple levels of hierarchy in the reductionist causal chain to shrink and explore the solution space of host-pathogen interaction. On a genome-scale, multi-cellular level, constraint-based hp modeling has great potential for the prediction of resultant physiologically perturbed cellular states. Implementation across these hierarchical levels of resolution (individual metabolites to mulit-cellular inter-species interactions) at several levels of abstraction will hopefully lead to further elucidation of the metabolic underpinnings of the acute and chronic process of infection, emergent mechanisms of pathogenesis, and therapeutic strategies to counteract such changes.

### Conflict of interest statement

The authors declare that the research was conducted in the absence of any commercial or financial relationships that could be construed as a potential conflict of interest.

## Notations/Abbreviations

h: a host model
p: a pathogen model
hp: a host-pathogen model
BM, h: host biomass pseudo-reaction
BM, p: pathogen biomass pseudo-reaction
S: the stoichiometric matrix for a metabolic network
v: flux vector in a metabolic network
x: metabolite vector in a metabolic network
m, the number of unique, compartment specific metabolites in a stoichiometric matrix, i.e.: |x|
n, the number of unique, compartment specific reaction fluxes in a metabolic network, i.e.: |v|
R: rank of the stoichiometric matrix
N_r_: size of the right null space
N_l_: size of the right null space
α: biomass optimum of host model
β: biomass optimum of pathogen model
ϵ: simulation constant for setting lower bound minimum of biomass production.
